# The impact of donor policies in Europe: a steady increase, but not everywhere

**DOI:** 10.1186/1472-6963-8-235

**Published:** 2008-11-13

**Authors:** Remco Coppen, Roland D Friele, Sjef KM Gevers, Geke A Blok, Jouke van der Zee

**Affiliations:** 1 NIVEL, Netherlands Institute for Health Services Research, PO Box 1568, 3500 BN Utrecht, The Netherlands; 2 Tranzo, Tilburg University, Tilburg, The Netherlands; 3 Department of Social Medicine, Health Law Section, Academic Medical Center/University of Amsterdam, Amsterdam, The Netherlands; 4 Faculty of Health Sciences, Department of Educational Development and Research, University of Maastricht, Maastricht, The Netherlands; 5 Faculty of Health Sciences, Department of Medical Sociology, University of Maastricht, Maastricht, The Netherlands

## Abstract

**Background:**

Transplantable organs are scarce everywhere. Therefore, countries have developed policies to support the efficient use of potential donors. Nevertheless, the shortage of organs remains. Were these policies in vain? The aim of this study is to assess the impact of donor policies on donor procurement in 10 Western European countries from 1995 to 2005.

**Method:**

To assess the impact of the donor policies we studied the conversion of potential donors into effectuated donors. 80% of the donors died from CVAs or a (traffic) accident. We considered these mortality rates to be a good proxy for potential donors. Here we call the conversion of potential donors into actual donors 'the donor efficiency rate by proxy'.

**Results:**

The mortality rates for CVA and (traffic) accidents have decreased in the countries under study. At the same time, in most countries the donor efficiency rates have steadily increased. The variance in donor efficiency rates between countries has also increased from 1995 to 2005. Four countries introduced a new consent system or changed their existing system, without (visible) long-term effects.

**Conclusion:**

The overall increase in donor efficiency means that the efforts to improve donor policies have paid off. However, substantial differences between countries were found. The success of donor policies in terms of the number of absolute donors is blurred by the success of policies on traffic safety and CVA treatment. It remains unclear which specific policy measures are responsible for the increase in donor efficiency rates. This increase is not related to having a presumed consent system. Furthermore, an analysis of countries that introduced a new consent system or changed their system showed no effect on donor efficiency.

## Background

Transplantable organs are scarce throughout the world. The discrepancy between the number of people listed on a national organ transplant waiting list and the number of post mortem organ donations per year results in long waiting times for patients to receive an organ [1,2]. In other words, the supply of organs that can be used for a transplant does not meet the need. The urgency of this problem was once again addressed by the EU Health Commissioner Markos Kyprianou in 2007 [3].

To deal with the scarcity of organs, countries have developed national organ donation policies. In most countries these donor policies consist of a legislative system which regulates consent for donation [4] and additional policy measures. Basically, there are two kinds of legislative systems: explicit consent and presumed consent. In an explicit consent system, the donor has to authorize organ removal after his death in the form of an advance directive or a codicil, or by registration in a national registry. If the deceased's wishes are unknown, next of kin are asked to consent. A presumed consent system does not require to obtain consent, either from the donor or from the next of kin; it is sufficient to verify that the deceased has not objected during his lifetime to becoming a donor [4]. In all countries, the group of non-registered residents is larger than the group of registered residents. Thus, presumed consent countries have larger pools of donor-consent than explicit consent countries, which might automatically result in greater numbers of donors in the former. Several authors have argued that differences in organ donation rates between consent systems prove that a presumed consent system leads to a more effective procurement of donors [5-7].

Other policy measures are directed at optimizing the process of donor procurement. Examples of such measures are hospital programs like Donor Action [8,9]. and EDHEP [10], or informing the public about the relevant aspects of organ donation [11,12]. To optimize the process of donor procurement, Spain developed an organizational model ('the Spanish model') with policy measures on national, regional, and hospital level to address the importance of organ donation and to effectively convert potential donors into actual donors [13-15].

In many countries these efforts have intensified over time. However, the shortage of organ donors remains. According to several studies [1,6,16,17]. the number of organ donations per million inhabitants (PMI) has increased only in some countries, whereas in most countries it has been stable or even decreased. Were these policy measures in vain? An international comparison of the performance of countries with regard to donor procurement over the years may provide some insight into the impact of policy measures in general.

To assess the impact of donor policies and to compare the performance of the different countries, a valid and reliable measure is needed. In many studies the national donation rates PMI are used. Several studies have demonstrated that using the number of donors PMI does not produce a valid comparison [1,18-30]. There are significant differences in the number of potential donors between countries, e.g. in the numbers of people dying from a CVA or (traffic) accident. Therefore, a measure which expresses the conversion of potential donors into effectuated donors PMI produces a more valid comparison than only the number of effectuated donors as a whole.

The aim of this study is to assess the impact of the donor policies in 10 Western European countries on donor procurement from 1995 until 2005. This study is part of the national evaluation of the Dutch Organ Donation Act [31].

## Method

### Population

To assess the impact of the donor policies we studied the conversion of potential donors into effectuated donors in 10 Western European countries. The number of confounding factors between countries was restricted by analysing only countries which share a more or less similar historical background and have more or less the same status of health systems.

### The number of potential donors per country

An exact measure for establishing a country's number of potential donors could be found by analysing all hospital medical records of deceased persons and identifying all potential donors [32-34]. Such data are not available for all countries. In most countries approximately 80% of donors die of a Cerebral Vascular Accident (CVA) or a traffic accident. A high correlation (Spearman's ρ = 0.81 (P < 0.01)) has been found between these mortality rates and donation rates [35]. In a study on differences between Dutch hospitals, Friele et al. also found this high correlation [34]. We therefore considered the national mortality rates for these causes of death to be a good proxy for the number of potential donors per country.

Another issue in establishing a country's number of potential donors is the selection of age groups. The number of people dying from a CVA increases with age, especially after the age of 65. Several countries implement senior donor programs [36,37]. However, except for Spain [23], the number of effectuated donations from donors older than 65 years is relatively small. The increase in mortality rates of CVA among people older than 65 does not lead to a matching increase in number of donors. The number of people dying from a CVA who are older than 65 years therefore seems to be of little relevance for a national proxy of the number of potential donors. In addition, a strong correlation is found between the mortality rates for CVA and traffic (accidents) among people younger than 65 and donation rates [35]. We therefore chose to restrict the proxy for the number of potential donors to the age groups below 65 years.

The data for CVA and (traffic) accident mortality rates were derived from the WHO's Health for All Database (HFA-DB) [38]. The age-standardized mortality rates in this international and uniform database are based on the International Statistical Classification of Diseases and Related Health Problems – 10^th ^Revision (ICD-10).

For some countries the mortality rates for certain years were missing in the WHO's HFA-DB. Because the mortality rates in all countries show a steady decrease we decided it was safe to estimate the missing mortality rates. The trend lines, which are based on estimated mortality rates, are shown in the graphs by a dotted line.

### The national organ donation rates

Different countries use different definitions for their national organ donation rates. To counteract these variations in national definitions we collected new data on the national number of donors based on one uniform definition. We asked the national transplant centres to send us their 'numbers of post mortal organ donors of whom at least one solid organ had been successfully transplanted per year'. This definition was preferred because it accounts for differences between countries in the quality of procured organs and it has a better coverage of the data in the period 1995 to 2005. Because France and Sweden could not provide their data according to this definition for the entire period (1995–2005), they were asked for their 'numbers of post mortal organ donors of whom at least one solid organ had been recovered for the purpose of organ transplantation [39]'.

From the countries that could provide the rates according to both ways of measuring organ donation rates we learned that the difference between both rates is no more than 5% overall. As we use the same definition within countries, the use of different definitions between countries does not affect the national trends for organ donation. Therefore, we found it acceptable to use the rates for France and Sweden, although they were obtained by using a deviating definition.

The rates per million inhabitants were calculated using the population size of the mid-year population given by the WHO HFA-DB [38].

### Analysis

In this study we assess the conversion of potential donors into actual donors by the donor efficiency rate by proxy. The donor efficiency rate by proxy is calculated by using the following definition: (national donation rates PMI/national mortality rates relevant for organ donation PMI) * 100. To determine significantly increasing or decreasing trends, the slopes of the donor efficiency trends of three time frames (1995–2005, 1995–1999, and 2000–2005) were calculated using a standard regression analysis.

### Consent systems

As several authors report a positive impact of consent systems on donor procurement [5-7]., we assessed the impact of consent systems on the donor efficiency rate. The information on the national consent systems is based on a legal analysis by Gevers et al. [4]. Table 1 shows information about the national consent systems and the changes which occurred in some countries during the period under review.

**Table 1:** Consent systems in 10 European countries (1995–2005)

**Consent systems (according to national legislation effective in 2005)**	
*Presumed consent*	Spain, Austria, Italy, France, Belgium, Sweden, the United Kingdom^1^
*Explicit consent*	Germany, the Netherlands, Switzerland
	
**Countries in which the legislative systems changed between 1995 en 2005**	
*Sweden*	On July 1 1996, Sweden changed from an explicit consent system to a presumed consent system. This change was accompanied by an information campaign to the Swedish public. All 4.2 million homes were informed about the system change and were neutrally motivated to take a stance in one or more of three ways: by telling next of kin, by signing a donor card, or by notifying the National Donor Register, established in 1996 [12].
*Germany*	On December 1 1997, the German Transplantation Act, in which an explicit consent system was laid down, came into force [4]. The passing of this Act was accompanied by a long and critical public debate, and several reports on organ donation in all media [50].
*The Netherlands*	In January 1998 the Netherlands laid down its explicit consent system in the Dutch Organ Donation Act. Along with the Organ Donation Act, a national donor registry (containing consents, refusals, or wishes that next of kin or specific person may decide) was implemented [4] and the Dutch Transplant Foundation was established. To accompany the introduction of the Organ Donation Act, the Dutch government supported neutral and soft-sell public campaigns [11]. Since 2000 the Dutch government has focused more on public recruitment campaigns and on supporting the process of organ donation in hospitals [34,51].
*Italy*	Italy enacted its new transplantation law in 1999, introducing a presumed consent system. The introduction of this new legislation was accompanied by the founding of a national transplantation centre in 2000 and improved organization of the donation process [52].

^1 ^According to the British Human Tissue Act of 1961 and the Human Organ Transplants Act of 1989 it is necessary to have consent of the donor to use his organs (explicit consent). However, when his will is not known it is (according to these Acts) sufficient to determine that the potential donor did not register an objection against organ donation. Consequently, the UK had a presumed consent system during the period under review [4,53]. By implementing the Human Tissue Act 2004 the UK introduced a formal explicit consent system in September 2006.

## Results: The organ donation rates and the national donor efficiency rates by proxy

**Figure 1:**
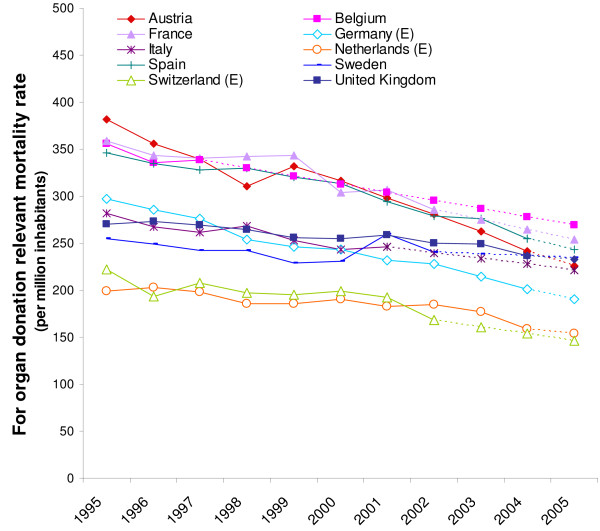
National mortality rates relevant for organ donation per million inhabitants in 10 European countries (1995–2005).

Figure 1 shows that there are differences between countries in the mortality rates relevant for donation. These rates have decreased during the last decade and have moved slightly towards each other.

**Figure 2:**
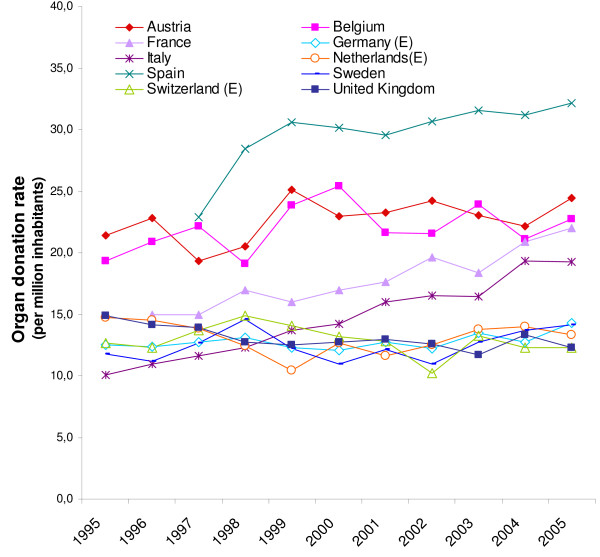
Organ donation rates per million inhabitants in 10 European countries (1995–2005).

Figure 2 shows the organ donation rates PMI in the 10 European countries.

There are large differences between the national organ donation rates PMI. As often mentioned in other studies, Spain has by far the highest rate per million inhabitants, followed by Austria and Belgium. These countries have twice as many organ donors PMI as for example Germany, the Netherlands, Sweden, Switzerland and the United Kingdom.

**Figure 3:**
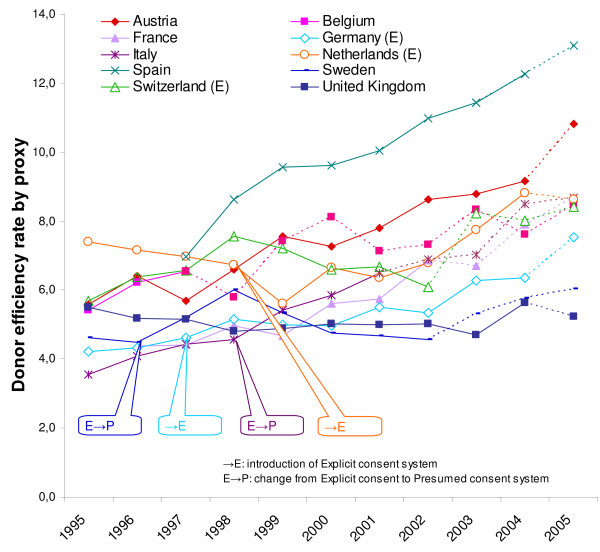
The donor efficiency rates by proxy in 10 European countries (1995–2005).

The impact of donor policies, taking into account differences in relevant mortality between countries, is demonstrated by the donor efficiency rates by proxy in figure 3. This figure shows that some countries have obvious trends in their donor efficiency rates. For Spain, Italy, France, Austria, and Germany the donor efficiency rates steadily increased from 1995 to 2005, whereas Switzerland, the Netherlands, Belgium, Sweden, and the United Kingdom have more fluctuating donor efficiency rates. Since the donor efficiency rates increased in some countries more than in others, the variance of donor efficiency rates between countries was larger in 2005 than in 1995.

Table 2 shows the slopes of the donor efficiency trends for three time frames (1995–1999, 2000–2005 & 1995–2005).

**Table 2:** Slopes of the donor efficiency trends in 10 European Countries (1995–2005, 1995–1999, and 2000–2005)

	1995–2005 *slope (ranking)*	1995–1999 *slope (ranking)*	2000–2005 *slope (ranking)*
Spain	0.682 (1)*	1.288 (1)	0.715 (1)*
Italy	0.512 (2)*	0.416 (3) *	0.577 (4)*
France	0.477 (3)*	0.144 (8)	0.620 (3)*
Austria	0.460 (4)*	0.413 (4)	0.632 (2)*
Germany (E)	0.279 (5)*	0.239 (7)*	0.463 (6)*
Belgium	0.262 (6)*	0.354 (5)	0.116 (9)
Switzerland (E)	0.198 (7)*	0.428 (2)*	0.436 (7)*
The Netherlands (E)	0.145 (8)	-0.402 (10)*	0.517 (5)*
Sweden	0.081 (9)	0.295 (6)	0.306 (8)*
The United Kingdom	-0.001 (10)	-0.162 (9)*	0.078 (10)
			
*Mean donor efficiency*	0.354*	0.269*	0.632*

* = significant increase/decrease

(E) indicates that this country has an explicit consent system

During the 1995–2005 period, most countries (Spain, Italy, France, Austria, Germany, Belgium and Switzerland) showed a significantly rising trend. In the same period the Netherlands, Sweden and the United Kingdom had no significant increase in their donor efficiency rates. We even see that during 1995–1999 the Netherlands as well as the United Kingdom had a significant decrease in their donor efficiency rates. The decreasing trend in the Netherlands recovered after 2000, showing a significantly rising trend instead. Likewise, the Swedish donor efficiency significantly increased in this period. In addition to figure 3, table 2 reveals that, on balance, the mean donor efficiency trend significantly increased during all three time frames.

On average, we did not find obvious differences between countries with a presumed consent system and countries with an explicit consent system (figure 3), nor did we find systematic differences between the slopes of the donor efficiency rates by proxy of presumed and explicit consent countries (table 2).

## Discussion

### The positive impact of donor policies on donor procurement

We found a decrease in relevant mortality rates (figure 1), which is likely to be due to the success of policies on CVA treatment and traffic safety. The strong correlation between the mortality rates for CVA and (traffic) accidents and donation rates [35,40]. means that policy measures on traffic safety and CVA treatment also have an impact on donor procurement [41]. For the procurement of donors the decrease in relevant mortality rates implies that it has become more difficult to find potential donors and that more effective strategies are necessary to prevent a decrease in organ donation rates.

After adjusting the organ donation rates for the changes in relevant mortality rates, most of the ten countries under review now demonstrate an increased efficiency in their donor procurement, implying a positive impact of donor policies in these countries. Not all countries show a steady increase in donor efficiency. The variance in donor efficiency rates by proxy increased from 1995 to 2005 (see figure 3). Apparently, the policies of some countries had more impact on donor procurement than others. The low impact of the donor policy in the United Kingdom has recently been addressed in an editorial by Smith & Murphy [42]. In this publication they report that the British Organ Donation Taskforce has recommended the implementation of a new framework for organ donation in the United Kingdom.

### Presumed consent versus informed consent

In our study we found no evidence that presumed consent systems perform better than explicit consent systems; we did not find obvious differences between consent systems (figure 3), nor did we find that donor efficiency rates by proxy of presumed consent countries increased in a more accelerated way than those of explicit consent countries (table 2).

From 1995 until 2005 some countries introduced a new formal consent system or changed their existing one (see table 1). These events often go together with mass media campaigns and changes in the organization of donor procurement. The causality between these events and their impact on donor procurement is difficult to prove. However, insight into these events may shed some light on our findings.

We see that with regard to the system changes in Germany and Italy there are no differences between the trends before and after the introduction of the (new) consent system. In both countries the donor efficiency trend was already increasing before the implementation of the new consent system and continued to increase in the same way after the introduction (see figure 3).

For Sweden and the Netherlands we do see differences between the donor efficiency trends before and after the introduction of their new consent system. The change to the Swedish system in 1996 led to a temporary increase in donor efficiency rate by proxy. In 1998 the Swedish donor efficiency reached the same level as before the introduction of their presumed consent system. Prior to the implementation of the Dutch explicit consent system in 1998 the donor efficiency trend was already decreasing, and one year after the introduction it reached its all-time low. Since 2001, the Dutch donor efficiency by proxy has increased. For the Netherlands we know that this probably has to do with the introduction of a range of new policy measures [31].

On the whole, none of the legal changes led to a significant change in the trend of a country's donor efficiency rate by proxy. It seems more likely that the changes in the donor efficiency trends in Sweden and the Netherlands were due to the impact of public awareness regarding organ donation.

Our findings to the effect that having a presumed consent system does not guarantee higher donation rates and that changing the consent system does not have a significant impact on the trends of the donor efficiency rates by proxy are in concordance with the findings of other studies. In a legal analysis of consent systems Gevers et al. point out that in reality the different systems are much more similar than suggested by the explicit/presumed distinction [4]. They furthermore conclude that in particular the predominant role of relatives, in case no decision of the deceased has been recorded (as is most frequent in both presumed- as well as explicit consent countries), reduces the potential of the presumed consent system. Several studies report the same finding that also in presumed consent practice the next of kin are consulted [14,43-46].

Our findings do not correspond with the findings of studies using national donation rates PMI to assess the impact of consent systems [5,6,47]. Abadie et al. conclude that, after controlling for other determinants for organ donation, presumed consent has a positive and sizeable effect on organ donation rates [18]. However, to control for differences in potential donors between countries they use mortality rates for all ages. By using CVA mortality rates of high age categories, it remains to be seen whether their method does not overestimate the contribution of CVA mortality rates to the donor potential.

### Limitations

A limitation of this study is that we cannot attribute the differences in donor efficiency rates by proxy between countries to specific policy measures. Nonetheless, according to our results, policies in general do have a positive impact on donor procurement. It is difficult to determine which specific policy elements are responsible for this success. There is no structured overview of specific donor policy measures per country, or of status of implementation regarding these measures per year. An exception to this general picture is the Spanish model, the effect of which has been described many times [13,14].

Another limitation of this study is that the numbers of potential donors are based on national mortality rates. Although the WHO puts a lot of effort into optimizing the reliability of the data, differences between countries in how they measure the mortality rates may occur. As a reaction to an editorial by Roels et al. [29], Matesanz et al. [48] conclude in their letter to the Editor that because of these possible differences, the number of donors PMI is the most realistic and simple way to make comparisons. However, neglecting huge differences in mortality rates between countries does not seem to be an option which leads to a valid international comparison. Neglecting differences in the number of, for example, traffic accidents will blur a valid comparison between countries regarding the impact of their donor policy. Besides, for the comparison between years within one country as well, we should take account of differences in the number of potential donors each year.

## Conclusion

This international comparative study shows that implementing policies for organ donation and putting effort into the procurement of donors does seem to have a positive impact on donor procurement in most countries. The success of donor policies is, however, blurred by the success of policies on traffic safety and CVA treatment and is therefore not revealed by the actual donation rates PMI.

Our results also demonstrate that in some countries donor policy had greater impact than in others. A gap is emerging between successful and less successful countries in this respect. Because there is insufficient information about which specific policy measures are implemented per country per year, it is not possible to determine which measures cause a significant increase in the donor efficiency rates by proxy.

The differences between countries and between years cannot be explained by differences in consent system between countries. Factors other than the consent system seem to be responsible for the differences between countries regarding the impact of their donor policy. These are for instance differences between countries regarding the measures they undertake to optimize the process of donor procurement (in hospitals), to increase the donor pool (by using older donors or non heart beating donors) or to inform the public about the relevant aspects of organ donation.

Whereas the shortage of donor organs persists, it is important to find pointers for the improvement of donor procurement. Because little is known about which specific policy measures are successful for each country, an organization like the European Commission, for instance, should support initiatives that achieve international comparable data on national policy measures among EU-members. In 2007 the European Commission organized two meetings to discuss organ donation and transplantation at EU level [49]. In addition, this study points out the necessity of adjusting such data for fundamental differences between countries (e.g. relevant mortality rates) which influence the organ donation rates. This can be encouraged by using a simple, yet effective method such as discussed in this study. Applying such a method will make it possible for policymakers to make evidence based decisions when implementing organ donation policy measures.

Nonetheless, policymakers should be aware of the fact that post mortal organ donation alone will not solve the long waiting times for patients in need of an organ. Therefore, other methods of donor procurement, such as encouraging living donation and the development of an artificial kidney, should be considered.

## Competing interests

The authors declare that they have no competing interests.

## Authors' contributions

RC is responsible for the study conception and design, carried out the data collection and drafted the manuscript. RDF is responsible for the study conception and design. All authors made substantional contributions to the analysis and interpretation of the data.

RDF, SKMG, GAB and JZ made critical revisions to the manuscript for important intellectual content and provided supervision. All authors read and approved the final manuscript.

## Pre-publication history

The pre-publication history for this paper can be accessed here:


